# The Temporal Context in Bayesian Models of Interval Timing: Recent Advances and Future Directions

**DOI:** 10.1037/bne0000513

**Published:** 2022-06-23

**Authors:** Renata Sadibolova, Devin B. Terhune

**Affiliations:** 1 Department of Psychology, Goldsmiths, University of London

**Keywords:** Bayesian timing, central tendency bias, migration effect, recency effect, dopamine

## Abstract

Sensory perception, motor control, and cognition necessitate reliable timing in the range of milliseconds to seconds, which implies the existence of a highly accurate timing system. Yet, partly owing to the fact that temporal processing is modulated by contextual factors, perceived time is not isomorphic to physical time. Temporal estimates exhibit regression to the mean of an interval distribution (*global context*) and are also affected by preceding trials (*local context*). Recent Bayesian models of interval timing have provided important insights regarding these observations, but questions remain as to how exposure to past intervals shapes perceived time. In this article, we provide a brief overview of Bayesian models of interval timing and their contribution to current understanding of context effects. We then proceed to highlight recent developments in the field concerning precision weighting of Bayesian evidence in both healthy timing and disease and the neurophysiological and neurochemical signatures of timing prediction errors. We further aim to bring attention to current outstanding questions for Bayesian models of interval timing, such as the likelihood conceptualization.

Although we are often aware of the relative inaccuracy of our subjective experience of the passage of time, we tend to be unaware of the factors that shape our temporal estimates. Human time perception is influenced and biased by a myriad array of factors. Two robust effects include temporal estimates showing regression to the mean of an interval distribution (*global context*; e.g., Acerbi et al., 2012; Jazayeri & Shadlen, 2010) and being affected by more immediate preceding trials (*local context*; e.g., de Jong et al., 2021; Wiener et al., 2014). Bayesian models of interval timing have provided important insights into these observations (Figure 1); however, it remains unclear how exposure to past intervals shapes perceived time at neural and mechanistic levels. In this article, we aim to provide a brief overview of how Bayesian models of timing contribute to our understanding of temporal context effects with an emphasis on recent research directions and outstanding questions.[Fig fig1]

The observation that perceived time is affected by the perception of previous intervals was first documented nearly two centuries ago (Fechner, 1948; Vierordt, 1868). The impact of the global context on interval estimates represents one of the best-known timing effects and is referred to as the Vierordt’s law, central tendency bias (CTB), or a migration effect across the vast literature on this topic. This effect describes the tendency for short and long stimuli among a set (or distribution) of intervals to perceptually migrate toward the mean and thus be over- or underestimated, respectively (Figure 1D; Acerbi et al., 2012; Cicchini et al., 2012; Jazayeri & Shadlen, 2010; Lejeune & Wearden, 2009; Petzschner & Glasauer, 2011; Shi et al., 2013). An abundance of studies have also reported local context effects (Figure 1E), known as *n* − 1 or recency effects, or time-order errors more generally. For instance, comparing two successive stimulus intervals may lead to an over- or underestimation of the second stimulus depending on factors such as the length of the interstimulus interval or the duration of the stimuli themselves (Burr et al., 2013; de Jong et al., 2021; Dyjas et al., 2012; Hellström, 1985; Nakajima et al., 1992; Wiener et al., 2014). Early theories explained these perceptual phenomena as a consequence of an adaptation and sensation weighting, with the latter defined as the utilization of “generic information” to supplement (uncertain) specific stimulus information (Anderson, 1971; Ellis, 1973; Hellström, 1985; Helson, 1947). Indeed, Bayesian and signal detection theories accounting for these perceptual biases figured in textbooks on cognitive psychology as early as in the 1970s (e.g., Hellström, 1985; Lindsay & Norman, 1977; Tversky & Kahneman, 1974).

These concepts were later expanded upon in the Bayesian decision theory (Cyert & DeGroot, 1987; Körding & Wolpert, 2006), which proposes that the brain may function as a Bayesian observer striving to optimally integrate prior knowledge and new sensory information using Bayesian inference (Friston, 2010; Knill & Pouget, 2004; but see Bowers & Davis, 2012). The general idea is that perception benefits from extracting statistical regularities from the environment that are modeled by a probability distribution (prior). In turn, incoming sensory data gives rise to the measurement distribution which specifies the likelihood function. Their integration results in the posterior distribution, which represents the perceptual decision space (Figure 1B). Here, a value is selected according to the implicit decision rule, determined by gains and losses specified in a cost function, which ultimately results in the response (Geisler & Kersten, 2002; Teufel et al., 2013). In Bayesian models of time perception, a perceived interval (posterior distribution) therefore results from a probabilistic inference including the internal temporal memory (prior) and new sensory evidence (likelihood; Acerbi et al., 2012; Gu et al., 2015; Rhodes, 2018; Shi et al., 2013). Priors and likelihoods may be represented by Gaussian probability distributions characterized by a mean and a variance, although more complex prior representations that incorporate higher order statistical features have also been reported (Acerbi et al., 2012).

According to Shi et al. (2013), the temporal prior may be centered on the mean of the presented interval range with the width of a distribution reflecting the precision of the internal memory reference (Figure 1D). The often-observed asymmetric regression to the mean may reflect the higher signal-to-noise ratio (narrower likelihood) for short durations, suggesting that the prior for an interval range distribution may form at a higher level of the processing hierarchy (van Rijn, 2016). By contrast, for the likelihood distribution, these parameters may characterize, respectively, the sensory readout and its precision for the *current* stimulus interval. Whereas priors and likelihoods superficially resemble the memory and internal clock (tick counts) components of the classic internal clock model (Gibbon et al., 1997; Shi et al., 2013), the difficulty of forming a continuous likelihood distribution given the discrete clock output has been noted in the literature (Rhodes, 2018). Shi et al. (2013) further identified as one of the outstanding questions in this domain the unification of a Bayesian timing framework with influential theories of time perception such as the striatal beat-frequency model. As will be discussed, one challenge for such efforts is almost universally implied poststimulus integration of the reference memory, whereas Bayesian temporal priors appear to shape the perception of ongoing stimulus intervals (Damsma et al., 2021).

The well-recognized strength of Bayesian models is their ability to account for context effects, including the influence of nontemporal factors such as general stimulus intensity or magnitude (Kruijne & van Rijn, 2021). Although earlier approaches combined a linear-weighted average and scalar timing theory in a “memory-mixing” model, they lack clarity regarding the mechanisms involved in subjective interval representation and the contribution of local and global contexts (Gu & Meck, 2011). By contrast, in Bayesian timing models (Jazayeri & Shadlen, 2010; Shi et al., 2013), temporal statistical regularities learnt rapidly through interval exposure lead to the formation of a prior that filters out the trial-by-trial noise in interval judgments. Put differently, the system treats each stimulus as reflecting both the stable properties of the world (that need to be inferred) and the uncertainty introduced, for example, through noisy sensory processing (that needs disregarding). However, by increasing the precision of temporal estimates this way, the model, however, inevitably introduces a systematic bias such as the CTB. Therefore, an identical stimulus interval is judged as shorter or longer depending on whether it is the longest or the shortest of a presented interval range, respectively (Acerbi et al., 2012; Jazayeri & Shadlen, 2010; Lejeune & Wearden, 2009). Taken together, Bayesian models provide a useful framework for, and novel insights into, timing phenomena such as the influence of global and local contexts. The subsequent sections will focus on more recent developments in the timing literature that further bear upon some outstanding questions for these models.

## Dynamic Integration of Multiple Priors

Despite numerous advantages of this framework, including its capacity to explain the CTB and account for performance patterns in special populations as will be discussed in later sections, the original Bayesian approach to timing assumed a relatively static prior (interval mean) throughout the course of an experiment (Acerbi et al., 2012; Jazayeri & Shadlen, 2010), thus failing to incorporate recency effects, which appear to impact temporal estimates independently of a global prior (de Jong et al., 2021; van Rijn, 2016; Wiener et al., 2014). Indeed, one of the outstanding questions that is seldom addressed by Bayesian timing models is how these multiple priors interact in shaping time perception. The internal reference model (Bausenhart et al., 2014, 2016; Dyjas et al., 2012) combines the global and local priors in a dynamically updated weighted geometric moving mean of earlier stimulus intervals. More recent Bayesian implementations include adaptive priors that change over time (Berniker et al., 2010; de Jong et al., 2021; Glasauer & Shi, 2021; Petzschner & Glasauer, 2011; Wiener et al., 2014) by also incorporating Kalman-like filters (Kalman, 1960). In these models, Kalman gain relates to the trial-by-trial precision (variance) of the prior and the likelihood distributions that affects their respective weights before they are integrated in a Bayesian optimal manner. For instance, low gain values may represent a scenario when a prior has stabilized over trials, whereas high values suggest a strong influence of the most recent trial. By applying such model, Wiener et al. (2014) demonstrated that despite their different timescales, both global and local context effects result from a common prior updated on a trial-by-trial basis. Moreover, recent implementation of a Kalman-like filter revealed that both the *range* and *sequence* of stimulus intervals determine the CTB (Glasauer & Shi, 2021). In a world that is relatively stable but fluctuates in small random walk changes, it stands to reason that a recent stimulus gives rise to a short-term expectation of the one that follows. It was shown that the CTB may be partially attributed to experimental randomization of stimuli, since it is substantially reduced for a random walk sequence (Glasauer & Shi, 2021). These results highlight the potential of modeling dynamic priors for elucidating how multiple priors may collaboratively shape time perception. Further, the inclusion of dynamic priors leads to more flexible and realistic theoretical models for how Bayesian priors may be acquired and used.

## Learning Priors

Prior formation represents a fundamental feature of Bayesian models of timing, yet our understanding of how different priors are learnt and how they generalize to different behavioral contexts is still limited. For instance, learning different statistical properties of a new prior may be differently paced as suggested by a more rapid learning of a prior mean compared to its variance (precision; Berniker et al., 2010; Miyazaki et al., 2005). Bayesian observers by default first appear to form a single prior by generalizing across sensory information, which serves to widen the data acquisition net and facilitate rapid learning (Roach et al., 2017). With extensive training, participants demonstrate a capacity to learn a large set of sometimes specific priors even if this may not be optimal. Roach et al. (2017) further showed that Bayesian observers can form multiple more specific priors when the corresponding interval distributions were coupled with multiple distinct motor outputs. This is consistent with the CTB being minimal for expert percussionists who show superior motor timing skills (Cicchini et al., 2012) and larger in Parkinson’s disease (PD), which is characterized by motor deficits (Malapani et al., 2002). Together with stimulus-specific priors learnt through more elaborate training, the structuring of prior knowledge is dynamic in the sense that the emphasis shifts from flexibility to specificity as learning progresses.

In another study, researchers applied functional magnetic resonance imaging (fMRI) in a time-to-contact estimation task to investigate the learning of across-trial regularities and trial-specific information (Polti et al., 2021). The caudate region signaled behavioral improvements specific to the stimulus interval in each trial. By contrast, hippocampus generalized across all stimulus intervals and its activation scaled with CTBs. Accordingly, a reduced influence of previous trials (local prior) on current reproduced intervals was observed after pharmacological silencing of hippocampal neurons (Dias et al., 2021), which implicates the hippocampal region in timing standardization by both temporal priors. These findings suggest that hippocampus and striatum facilitate two distinct forms of learning, supporting, respectively, the generalization and specificity in time perception in a rapid manner. Another recent study in the nontemporal domain showed that generalization strength is related to functional connectivity between the dopaminergic midbrain and hippocampus (Kahnt & Tobler, 2016). Further, a hippocampal dopamine D2-receptor blockade by amisulpride led to decreased midbrain–hippocampal connectivity and altered generalization gradients. Altogether, these results implicate the hippocampal–striatal network in generalization by temporal priors, and they suggest that rather than fixed and “hard-wired,” generalization may be flexible and it can be modulated by pharmacological intervention (Kahnt & Tobler, 2016).

Further research into the role of the striatal–hippocampal interplay is thus required to understand the learning of temporal priors and the generalizability and specificity in timing performance. According to the internal reference model, which bears similarity to Kalman filter models, internal references may not be necessarily replaced by more current ones (Bausenhart et al., 2016). Instead, they may be retained to some extent and used to reconstruct the original reference (Ogden et al., 2008) and in some situations, a long-term reference perseveres in the face of a more current temporal context (Wearden, 2008). A further outstanding question is how these effects manifest in long-term training of interval timing (Bueti et al., 2012).

## Temporal Locus of Prior Influence During Temporal Processing

The integration of temporal priors and sensory evidence is implicitly reserved for the postsensory phase of the processing of temporal intervals, given that the duration of a temporal stimulus (likelihood) is ostensibly available only once the stimulus has ended (Damsma et al., 2021; Gibbon, 1977; Matell & Meck, 2004). In some cases, temporal decisions may precede the offset of (long) stimulus intervals in tasks such as temporal bisection, once the duration of a subjective mid interval (internal reference) is surpassed (Balci & Simen, 2014). Electroencephalography (EEG) can capture brain dynamics in high temporal resolution and is therefore suitable for investigating the stage during temporal processing when temporal priors and likelihoods are integrated. Past research has shown that larger amplitude of the contingent negative variation (CNV) and increased beta oscillatory power associated with longer *n* − 1 prior durations indeed reflect the adjustment of a decision threshold in drift diffusion modeling (Wiener et al., 2018). However, it was later suggested that these EEG signatures may not unambiguously reflect the integration of priors in the decisional stage due to the nature of the temporal bisection task (Damsma et al., 2021). Rather, they may reflect the updating of the internal reference on a trial-by-trial basis.

Most models of interval timing have largely assumed that integration with memory (priors) occurs in later perceptual stages. By contrast, relatively little attention has been devoted to the possibility that temporal priors may shape perception by modulating stimulus interval processing online. However, recent evidence suggests that this may indeed be the case. Electrophysiological evidence suggests that temporal priors shape perception of an interval during stimulus processing as well as in the postsensory stage (Damsma et al., 2021; Sohn et al., 2019). Damsma et al. (2021) methodically explored the EEG correlates of both global and local context effects in a temporal reproduction task, implicating the CNV, P2 amplitude and latency and β-band power over fronto-central electrodes, and the distributed neural code during the encoding of stimuli in human participants. The authors suggest that neural populations associated with the CNV may perform temporal scaling based on context (Remington et al., 2018; Sohn et al., 2019), pointing out that they were able to decode the context from EEG dynamics almost instantaneously following stimulus onset. Their observations concerning the P2 component were related to expectancy suggesting active interval anticipation based on previous stimuli. Finally, they showed an association between the prior and β-band power during the currently processed stimulus interval, earlier than in the decisional stage as was previously thought (Wiener et al., 2018).

Another electrophysiological study involving saccadic and motor temporal production in two monkeys explored Bayesian integration at the level of single neurons as well as population networks (Sohn et al., 2019). It was found that temporal priors affected behavior through the modulation of latent dynamics in frontal cortex. Computational modeling was used to show how priors may establish a curved trajectory in neural space. Neural states would be projected along this trajectory onto an encoding axis, creating a warped time representation reflecting the influence of priors. Taken together, these studies suggest that temporal priors may shape time perception earlier than was previously thought, during the ongoing processing of a stimulus interval thus diverging from earlier theories that tended to incorporate sequential order of the sensory processing and memory stages (Gibbon, 1977; Jazayeri & Shadlen, 2010; Matell & Meck, 2004).

## The Temporal Likelihood Conundrum

**Figure 1 fig1:**
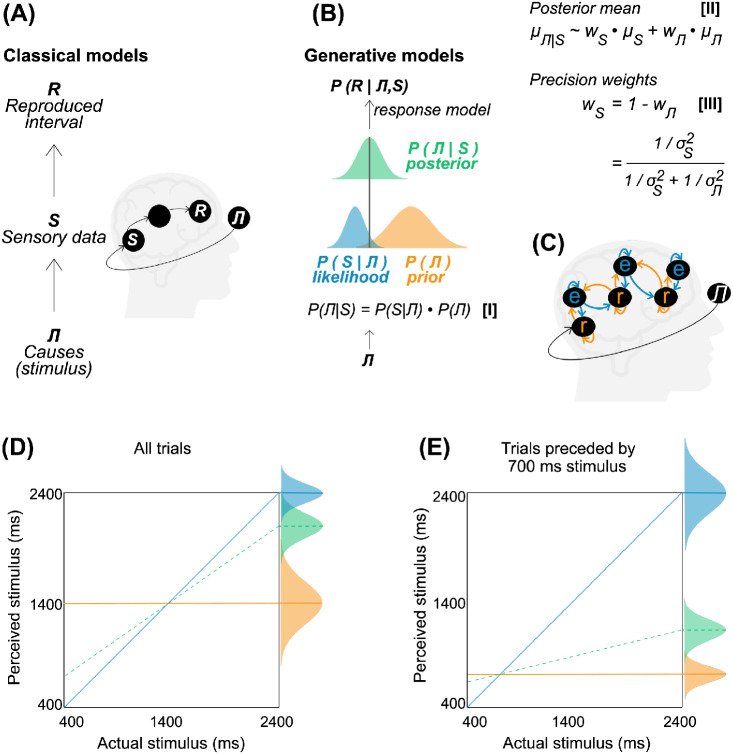
Temporal Models and Biases *Note*. (A) In classical models, the causes of sensory stimulation are not predicted. The sensory information ascends through levels of a processing hierarchy gaining on complexity until it translates into a decision (e.g., perceived time). (B) In generative models, a joint probability of these causes and sensory data is probabilistically inferred with Bayes rule (*Equation I*; Petzschner et al., 2015). Importantly, the resulting posterior mean estimate is shaped by the precision (inverse of variance) of the prior and likelihood distributions. It is the uncertainty-weighted average of the prior mean and the likelihood mean (*Equation II*) with their precision weights being inversely proportional to their respective variances (*Equation III*). The panel (C) depicts how the top-down (orange) and bottom-up (blue) chains interact in hierarchical predictive coding. The orange arrows and the nodes with letter “*r*” represent predicted neural responses (priors), whereas the blue arrows and the nodes with “*e*” represent a mismatch (error) between the predicted and actual neural responses (likelihood). (D–E) An illustrative example of the global and local context effects (D and E panels, respectively) in perceived-by-actual interval plots. The measurement of a stimulus interval is represented by a likelihood function (in blue). Both panels show deviations in perceived intervals (posterior; in green) toward the prior (in orange), that is, the mean of stimulus interval range (global prior) or the preceding 700-ms stimulus (local prior). The plots further show how the lower (D) and higher (E) prior precision relative to the precision of a likelihood impact on the magnitude of temporal bias. For the former, the responses are closer to the likelihood and therefore less biased, whereas they are significantly biased in the case of a latter. If participants responded only with a prior, their responses would fall on the orange lines. By contrast, veridical temporal estimates reflecting no prior influence would fall on the blue line. See the online article for the color version of this figure.

Rather than dealing with a bottom-up hierarchical accumulation of stimulus characteristics (Figure 1A), predictive processing models assume that perceptual systems invert the structure of learning about the world by applying and testing hierarchical generative models (Figure 1B). The incoming signal representations and their precision are thus predicted top-down at each processing stage and the incoming information is used as training data to fine-tune these predictions (Figure 1C). As such, these models suggest that perceptual systems combine two powerful processing features: The subtracting away of the signal predicted by priors by way of confirming the predictions of a generative model and the selective amplification of unexplained incoming information that represents uncertainty (Clark, 2013; Friston & Kiebel, 2009; Hohwy, 2012; Shi & Burr, 2016). Notably, since the predicted signal is subtracted away, the likelihood at each level of the processing hierarchy is reduced to the unexplainable signal (*prediction error*) and as such it is relayed on. Henceforth conceptualized as prediction errors, the likelihood can be modulated by attention-like processes at each level in the hierarchy (Figure 1C). Specifically, attention has been likened to a selective increase of gain on those prediction errors that fast-track the improvement of subsequent predictions and thus more efficiently reduce uncertainty (Clark, 2016).

This view of a likelihood does not conform to some conceptualizations of the temporal likelihood in the Bayesian timing literature, such as the output pulse count of a dedicated internal clock, ramping neural activity, or some other interoceptive process (Kononowicz & van Rijn, 2014; Rhodes, 2018; Richter & Ibáñez, 2021; Shi et al., 2013). However, recent developments have shown that the capacity to explain how a Bayesian observer perceives time may not be contingent on the output of a putative internal clock. On the contrary, a network representing the visual system was able to produce reliable interval estimates that were similar to human timing judgements simply from the accumulation of salient changes in to-be-timed real-life video stimuli (Roseboom et al., 2019). Specifically, rich dynamic stimuli were perceived as shorter when fewer salient changes transpired in the course of their duration, demonstrating that the implicit processing or awareness of changes in perceptual content alone may account for the perception of duration. This model is conceptually similar to neural population “clocks” (Karmarkar & Buonomano, 2007; Paton & Buonomano, 2018; Sadibolova et al., 2021), which assume that timing is an intrinsic property of neural networks, with the temporal stimulus dimension modeled as a trajectory in a neural state space (see also Matthews et al., 2014).

Recently, a Kalman filter was suggested to model Bayesian predictive processing with forward prediction errors as the temporal likelihood (Shi & Burr, 2016). This account assumes a twofold corrective process for minimizing prediction errors that involves the updating of a posterior distribution (percept) and/or dynamic adjustment of a prior for more reliable future predictions (for evidence in support of both optimal and suboptimal Bayesian integration in this framework, see Shi & Burr, 2016). By way of example, the strength of regularization by priors correlates with temporal Weber’s fractions, showing that priors rapidly lose influence as sensory uncertainty decreases (Burr et al., 2013). When incoming sensory information is reliable, a less precise generative model producing poor predictions requires recalibration to better fit the external world. This has been shown to be the case in studies on sensory-motor recalibration (Stetson et al., 2006; Vercillo et al., 2015; Vicario et al., 2016), further demonstrating that action is one of the potent calibrators of internal time representations. Taken together, the ambiguity regarding the temporal likelihood touches on a long-standing controversy in the field pertaining to the existence of an internal clock versus clock-free timing (Matell et al., 2006; Paton & Buonomano, 2018) and as such, it may bear diverse forms of Bayesian model implementation.

## The Precision of the Likelihood and Prior Distributions

To form a more reliable percept of the environment, the brain needs to estimate sensory uncertainty (Beierholm et al., 2020). For instance, the magnitude of the CTB is related to the magnitude of uncertainty in sensory measurement and the select cost function (Mamassian & Landy, 2010). Deteriorating precision (variance) of the Bayesian likelihood compensated for by an overreliance on temporal priors is thought to account for a larger CTB in older adults (Turgeon et al., 2016). Increased noise and temporal uncertainty together with impairments in attention and memory that come with normal aging likely mediate these timing impairments. Conversely, expert percussionists exhibit a reduced CTB relative to other musicians and controls, reflecting elevated precision weighting of sensory information presumably due to their extensive interval training (Cicchini et al., 2012). Similar observations are made in clinical populations. For instance, patients with PD show a greater CTB than controls and yet further CTB magnification upon withdrawal of their dopamine medication (Gu et al., 2015; Malapani et al., 2002). Their CTB is reduced both with learning through feedback and with dopamine agonists, which points to the partly overlapping dopamine-mediated mechanisms for learning and signaling of perceptual likelihood precision (Gu et al., 2015). Further, the pathogenesis of autism spectrum disorder is associated with a deficient dopaminergic system (Pavăl, 2017) and children with autism do not optimally use Bayesian temporal priors to reduce errors in temporal reproduction tasks (Karaminis et al., 2016).

However, assuming that the CTB scales monotonically with lower precision of the likelihood and low tonic dopamine levels (Gu et al., 2015; Mikhael et al., 2021) may be an oversimplification given apparent contradictory evidence for an increased performance standardization by temporal prior with elevated tonic dopamine on amphetamine and in schizophrenia (Cassidy et al., 2018). Hallucinations in schizophrenia have been attributed to the breakdown of perception with the false generation of prediction errors and their high precision weighting (likelihood) as well as the breakdown in top-down beliefs (priors; Fletcher & Frith, 2009). Although dopamine was previously suggested to signal the salience of prediction errors by encoding the precision of a likelihood distribution (Friston et al., 2012), it has also been associated with the precision weighting of Bayesian *priors* in schizophrenia. A recent temporal reproduction study (Cassidy et al., 2018) aimed to investigate the precision signaling of temporal priors in patients with schizophrenia given their pronounced timing deficits (Thoenes & Oberfeld, 2017). Cassidy et al. (2018) manipulated in each trial the precision of the prior distribution (expectations formed from a sequence of 2–4 context intervals) before asking patients and controls (on and off amphetamine) to reproduce a fixed auditory target interval (700 ms) that followed. The auditory context tones formed a 2 by 3 design being drawn from a distribution with low or high variance and short, medium, and long means (relative to the target interval). Both groups exhibited a clear CTB in the low-variance condition, suggesting that the uniform context intervals produced a narrow (precise) interval prior that was subsequently up-weighted in their reproductions. By contrast, only the patients and controls on amphetamine exhibited the CTB in the high-variance condition. This suggests that elevated dopamine augments prior precision weighting under uncertainty.

In an independent line of research, the dopamine antagonist haloperidol has been shown to impair temporal expectations and disrupt signaling of an otherwise precise temporal prior (Tomassini et al., 2016, 2019). In separate experiments, patients with PD and healthy participants were presented with foreperiods between *warning* and *go* stimuli, at which point they were required to respond as quickly and accurately as possible by pressing a key. The foreperiod intervals were drawn from high- or low mean distributions with high or low variance. Critically, the low-variance conditions resulted in fast learning and higher predictability of the expected foreperiod offset and concomitantly faster response times. The administration of haloperidol strikingly reduced this advantage of a precise prior, independently of a motor impairment (Tomassini et al., 2016). Taken together, these observations highlight that dopaminergic dysregulation may be associated with disrupted precision signaling for both priors and likelihoods.

Although dopamine has long been a focus of research in the study of interval timing (Coull, Cheng, et al., 2011), it is unlikely to be the sole neuromodulator that signals the precision of Bayesian evidence and therefore uncertainty. Other neurotransmitters have been implicated in associative learning tasks in nontemporal domains, such as acetylcholine (Iglesias et al., 2021) and noradrenaline (Lawson et al., 2021). For instance, preliminary data suggest that whereas low-level sensory prediction errors activate the dopaminergic midbrain, high-level sensory prediction errors about the probability of the sensory outcome activate the cholinergic basal forebrain (Iglesias et al., 2021). The distinct dopamine–acetylcholine mechanisms are yet to be confirmed, however, since this interpretation is complicated by the fact that the midbrain receives cholinergic inputs while basal forebrain receives dopaminergic inputs as well as additional GABAergic and glutamatergic connections in these regions. In another study, the administration of propanol (noradrenergic blockage) resulted in increased overconfidence in one’s priors and diminished learning of cue–outcome contingencies (Lawson et al., 2021). Elsewhere it has been argued that classic psychedelics, which are known to modulate interval timing (Wittmann et al., 2007; Yanakieva et al., 2019) and primarily operate on the 5-hydroxytryptamine 2A (5-HT2A) receptor as partial serotonin agonists, may produce their psychoactive effects by reducing the precision of priors high up in the cortical hierarchy (Carhart-Harris & Friston, 2019). Finally, in the predictive coding framework, unexplained prediction errors are thought to be signaled by α-amino-3-hydroxy-5-methyl-4-isoxazole-propionic acid (AMPA) receptors at ascending connections of a processing hierarchy, whereas the predictions descend top-down at N-methyl-D-aspartate (NMDA) receptors (Self et al., 2012). Ketamine, which has been shown to reliably modulate interval timing (Cheng et al., 2007; Coull, Morgan, et al., 2011) and has been proposed as a pharmacological model of schizophrenia (Corlett et al., 2019), has been shown to reduce the electrophysiological signature of prediction errors (mismatch negativity) by inhibiting NMDA receptors and thus disrupting the inference of statistical regularities (Coull, Morgan, et al., 2011; Weber et al., 2020).

## Temporal Decisions

In predictive processing, the unexplained signal (prediction error) travels to another processing level in the cortical hierarchy and the incoming signal is only used for tuning and nuancing predictions rather than encoding the state of the world. Yet, the perception of the stimulus is not contingent on eliminating *all* forward-flowing prediction errors. Indeed, Clark (2016) proposes a piecemeal succession from a perception of a general nature (“gist”) toward an increasingly richer percept as the residual errors gradually decrease (Clark, 2016; Hegdé, 2008). In accordance with this hypothesis, recent timing data showed a reduced regression to the mean in reproduced intervals (i.e., smaller CTB) when participants had an opportunity to respond a second time immediately after their first response with or *without* feedback (Bader & Wiener, 2021). Given that temporal priors shape perception of ongoing stimulus intervals (Damsma et al., 2021; Sohn et al., 2019), it is perhaps not surprising that the first response reflects the CTB more than the second response. Moreover, the first response may allow participants to evaluate their performance and incorporate new information into subsequent temporal decision-making.

Bayesian models concerned with a “pure” expression of a posterior distribution resulting from prior and likelihood integration would be incomplete without perceptual decisions reflecting both the posterior beliefs as well as the expected cost (loss function; Feldman, 2014; Jazayeri & Shadlen, 2010; Shi et al., 2013). In Feldman (2014) illustrative example, the posterior belief in two hypotheses (heart attack or heartburn) would not be sufficient in decision-making without considering the consequences of misclassifying the heart attack (patient’s death) and heartburn (unnecessary medical procedures). Bayesian decision theory therefore assumes that final timing responses will reflect the best posterior distribution estimate that additionally maximizes the benefit to the observer. To help with their responses, participants must therefore develop expectations about the relative cost of correct and incorrect responses. Indeed, the evidence shows that cognitive apparatus monitors endogenous performance uncertainty to guide timing decisions and that human participants are, to a certain extent, aware of the direction and magnitude of their temporal errors (Akdoğan & Balci, 2017; Balci et al., 2009, 2011; Foote & Crystal, 2007; Simen et al., 2011). Moreover, Bader and Wiener (2021) showed that participants may use this internal error monitoring to reduce the CTB, and that they further benefit from reduced timing uncertainty (coefficient of variation) when external performance feedback is provided. Furthermore, reinforcement learning compensates for detrimental slow memory drifts that introduce timing variability, even when maximizing the reward exploration in itself also increases variability (Wang et al., 2020). Conversely, timing choices under conditions of high uncertainty show a preference for safe (low cost), albeit small, rewards and uncertainty about how long the reinforcement is available leads to vigorous performance to maximize the reward benefit (Balci et al., 2011; Foote & Crystal, 2007). Altogether this evidence suggests a close link between the processes underlying time perception and learning.

Several attempts have recently been made to consolidate mostly independent work on the roles of dopamine in time perception and reward prediction learning (Fung et al., 2021; Mikhael et al., 2021; Mikhael & Gershman, 2019; Petter et al., 2018; Toren et al., 2020). Although both predictive processing and reinforcement learning models are preoccupied with prediction errors, their focus appears to differ. Whereas the former strives to minimize perceptual prediction errors (surprise and uncertainty), the latter builds on maximizing the rewards. Referring to rational inattention in behavioral economics, Mikhael et al. (2021) introduced a model that asserts that increasing task precision (e.g., improving temporal performance) is associated with higher energetic costs. Whether the agent decides to incur this cost depends on tonic dopamine. For example, high dopamine under high average reward availability increases learning from positive reward errors and tips behavior toward exploitation (Gershman & Tzovaras, 2018). In this scenario, temporal priors are thought to have diminished influence, whereas the likelihood precision increases. Conversely, low dopamine is associated with exploration and learning from negative reward prediction errors, as well as an increased CTB and lower temporal likelihood precision. Although this model accounts for a larger CTB in PD as well as its reduction in response to pharmacological intervention (Gu et al., 2015; Malapani et al., 2002), it cannot account for a larger CTB with elevated dopamine in schizophrenia or under amphetamine (Cassidy et al., 2018). The relationship between dopamine, timing, and reinforcement learning may not follow a monotonic linear function (Linnet et al., 2012; Wallace et al., 2011) and it may be more complex. Further research is required to disentangle these ostensible inconsistencies.

## Summary and Future Directions

This work has sought to provide a synthesis of recent research aiming to elucidate interval timing from the perspective of Bayesian inference. Bayesian inference has been successfully applied to account for well-documented interval timing biases such as recency and migration effects (Acerbi et al., 2012; Cicchini et al., 2012; Jazayeri & Shadlen, 2010). Recent evidence argues against static Bayesian priors (de Jong et al., 2021; Glasauer & Shi, 2021) and we are beginning to understand the specifics of prior formation (Polti et al., 2021; Roach et al., 2017). A Bayesian prior does not only represent the range of intervals (prior distribution mean and variance) but also their sequence (Glasauer & Shi, 2021), which has not been historically considered. Further work will also be required to disentangle precision weighting of the prior and likelihood and in particular the role of dopamine in these processes. Future research will similarly benefit from clarifying how Bayesian timing is instantiated within independent neurochemical systems or complex multisystem (e.g., dopamine–glutamate) interactions. There are numerous outstanding questions regarding the utility of the Bayesian inference framework in the study of interval timing. In particular, future research is warranted in the consideration of discrepancies in the conceptualization of temporal likelihoods. For example, is it an output of a dedicated timing system or a property of neural networks (Matell & Meck, 2004; Paton & Buonomano, 2018; Roseboom et al., 2019)? How can Bayesian models of interval timing be reconciled with competing accounts, such as those based on an internal clock, coincidence detection, or neural networks? To the extent that these models are incompatible, it will be imperative to draw out their divergent predictions in order to facilitate adversarial tests of these accounts, which should engender further updating and potential rapprochement between rival theoretical camps. Taken together, the principal strength of Bayesian inference is in optimal integration of prior knowledge and new sensory information that offers insights into perceptual processes. Determining the extent and limits of these insights continues to be debated.
